# Relationship of natural killer-cell activity to rhesus antigens in man.

**DOI:** 10.1038/bjc.1979.46

**Published:** 1979-03

**Authors:** P. Hersey, A. Edwards, C. Trilivas, H. Shaw, G. W. Milton

## Abstract

A number of previous studies have shown that the level of natural killer (NK) cell activity in humans is relatively constant for a given individual but varies widely between individuals. The factors which determine this variability are largely unknown, but genetic factors appear to be involved. In the present study it was found that Rh- normal subjects and melanoma patients had significantly higher natural cytotoxicity to target cells than Rh+ patients. This difference did not appear to be due to sensitization against Rh antigens on the target cell and may indicate that genes determining NK-cell activity are associated with those determining the expression of Rh antigens. Analysis of the survival data for Rh- and Rh+ patients did not reveal any increase in survival attributable to the higher natural cytotoxicity in Rh- patients.


*Br. J. Cancer* (1979), **39**, 234

### RELATIONSHIP OF NATURAL KILLER-CELL ACTIVITY TO RHESUS ANTIGENS IN MAN

P. HERSEY, A. EDWARDS, C. TRILIVAS, H. SHAW AND G. W. MILTON

*From the Kanematsu Memorial Institute and Melanoma Unit, Department of Surgery, University of Sydney, Sydney Hospital, Australia*

Received 11 September 1978 Accepted 27 November 1978

**Summary.—A number of previous studies have shown that the level of natural killer (NK) cell activity in humans is relatively constant for a given individual but varies widely between individuals. The factors which determine this variability are largely unknown, but genetic factors appear to be involved. In the present study it was found that Rh$^-$ normal subjects and melanoma patients had significantly higher natural cytotoxicity to target cells than Rh$^+$ patients. This difference did not appear to be due to sensitization against Rh antigens on the target cell and may indicate that genes determining NK-cell activity are associated with those determining the expression of Rh antigens.**

**Analysis of the survival data for Rh$^-$ and Rh$^+$ patients did not reveal any increase in survival attributable to the higher natural cytotoxicity in Rh$^-$ patients.**

CERTAIN MONONUCLEAR CELLS from the blood of normal human subjects, referred to as natural killer (NK) cells, have the ability to kill a variety of cultured tumour cells *in vitro* (Pross & Baines, 1977; Keissling & Haller, 1978; Hersey, 1979). The nature of NK cells is still controversial, although many workers agree they are neither B lymphocytes nor macrophages. Recent studies by West *et al.* (1977) have supported the notion that they may be a sub-class of T lymphocytes (Hersey *et al.*, 1975) but the question is far from settled.

The mechanism of killing by NK cells is uncertain, and early evidence suggesting that it is an antibody-mediated mechanism (Akira & Takasugi, 1977) has not yet been widely confirmed (Bonnard *et al*, 1979). Trinchieri & Santoli (1978) have recently shown that interferon is involved in the generation of NK-cell activity, and it is possible that interferon or lymphotoxin-like factors may be involved in the cytotoxic mechanism (Bonnard *et al.*, 1979; Peter *et al.*, 1976).

It is generally accepted that, although the level of NK-cell activity is constant for a given person from day to day there is wide variation in activity between individuals. The factors underlying this variation are poorly understood. It has been shown in mice that the levels of NK-cell activity vary between different strains, and is an inherited characteristic linked to $H_2$ genes (Kiessling *et al.*, 1975; Keissling & Haller, 1978). Several studies in humans also suggest that NK-cell activity may be under genetic control. Petranyi *et al.* (1974) found that mononuclear cells from HLA A3B7 subjects had low NK-cell activity to xenogeneic target cells. These findings were confirmed and extended by Santoli *et al.* (1976), who also found male subjects to have higher NK-cell activity than females. In the present report we present evidence, from studies on normal subjects and melanoma patients, that inheritance of Rhesus (Rh) antigens may also be involved in the regulation of NK-cell activity of human subjects.

Correspondence to: Dr P. Hersey, Medical Research Department, Kanematsu Memorial Institute, Sydney Hospital, Sydney, N.S.W. 2000, Australia.

### MATERIALS AND METHODS

*Normal subjects.*—Blood samples were taken from 80 normal subjects (48 male, 32 female) who were volunteer blood donors. Their ages ranged from 19 to 63 years. Mean ages were 36 for males and 31 for females. Forty (20 females, 20 males) were positive for Rhesus D antigens ($Rh^+$) and 40 (12 females, 28 males) were negative for Rhesus D antigen ($Rh^-$). Studies on these subjects were carried out on 4 separate days.

*Melanoma patients.*—The results of assays on 95 $Rh^+$ and 18 $Rh^-$ male patients and 80 $Rh^+$ and 23 $Rh^-$ female patients over the period 1977–78 were included in the study. Most of the assays were on patients who had surgery to remove primary melanoma, with or without regional lymphnode dissection (Stage I & II) (73 $Rh^+$ and 13 $Rh^-$ males, 59 $Rh^+$ and 16 $Rh^-$ females). Some of the assays were on patients with disseminated melanoma Stage III, who had palliative removal of subcutaneous nodules (21 $Rh^+$ and 5 $Rh^-$ male patients, 21 $Rh^+$ and 6 $Rh^-$ female patients). Many of the patients were treated with BCG vaccination with or without chemotherapy with imidazole carboxamide, 2–4 weeks after removal of their melanoma. There was no bias towards any particular form of treatment in $Rh^+$ or $Rh^-$ patients.

### *$^{51}Cr$-release assay*

Assays of NK-cell activity were carried out essentially as described previously (Hersey *et al.*, 1978).

Effector cells were obtained from defibrinated venous blood samples by centrifugation on Hypaque: Ficoll mixtures as described by Böyum (1968). They were resuspended in RPMI+10% foetal bovine serum (FBS) at a concentration of $6\times10^5$/ml.

Target cells were (1) Chang cells from long-term tissue culture (Commonwealth Serum Laboratories, Melbourne) and (2) melanoma cells from the MM200 line described previously (Hersey *et al.*, 1976). The cells were harvested by incubation in 0·25% trypsin for 15 min and labelled with $^{51}Cr$ by incubation with 100 $\mu$Ci $Na_2{}^{51}CrO_4$ (Amersham, Bucks, U.K.) at 37°C for 2 h. They were washed twice in 30 ml of Hanks' balanced salt solution and resuspended at $6\times10^3$/ml in RPMI+10% FBS.

Target cells (0·5 ml) and effector cells (0·5 ml) were incubated together in duplicate 10×70 mm round-bottomed tubes for 16 h. They were then harvested by centrifugation at 400 *g* for 7 min and 0·5 ml supernatant withdrawn for counting. The tubes were counted in a gamma counter and percent $^{51}Cr$ release calculated by the formula:

$$\%^{51}\mathrm{Cr\ release} = \frac{2a}{a+b} \times 100$$

Where $a$=ct-background in tube containing the supernatant only and $b$=ct-background in the tube with the cells and remaining supernatant.

*Statistics.*—The significance of the difference between the means of the NK-cell activity values of effector cells from the $Rh^-$ populations were determined by Student's *t* test. The difference in the survival rates of the $Rh^-$ and $Rh^+$ patients were determined by logrank analysis of the data (Peto *et al.*, 1977).

### RESULTS

### *NK-cell activity values of $Rh^-$ and $Rh^+$ normal subjects*

The NK-cell activity values of 40 normal $Rh^+$ and 40 $Rh^-$ subjects are shown against Chang cells and the melanoma cells from the MM200 cell line in terms of $^{51}Cr$ release above the baseline of $^{51}Cr$ release from target cells alone. (For

FIG. 1.—NK activity of $Rh^+$ and $Rh^-$ normal subjects against MM200 melanoma cells and cultured Chang "liver" cells. Mean percent $^{51}Cr$ release (±s.d.) for $Rh^+$ subjects against Chang and MM200 were 13·2±5·3 and 12·2±5·4 respectively and for $Rh^-$ subjects 16·5±7·1 and 15·9±8·7.

Chang cells the baseline $^{51}Cr$ release in the 4 consecutive experiments was 32, 35, 32 and 34%. For the MM200 target cells the $^{51}Cr$ release in these experiments was 24, 38, 40 and 36%. One value of an $Rh^-$ subject against the MM200 was lost due to technical error.)

A difference in distribution of the NK-cell values of $Rh^-$ and $Rh^+$ subjects were seen in the histograms (Fig. 1). The arithmetic means $\pm$s.d. of NK-cell activity from $Rh^-$ and $Rh^+$ subjects against MM200 cells were $15·9 \pm 8·7$ and $12·2 \pm 5·4$ respectively ($0·01 < P < 0·0125$). The mean NK-cell values against Chang cells were $16·5 \pm 7·1$ and $13·2 \pm 5·3$ respectively ($0·005 < P < 0·01$). There was no significant sex difference between NK-cell values within each group against either target cell.

*NK-cell activity values of $Rh^-$ and $Rh^+$ melanoma patients*

NK assays against MM200 and Chang cells were conducted on melanoma patients before, and 2–4 weeks, 2–3 months and 4–6 months after surgical removal of melanoma. The mean values of 2–4 assays

FIG. 2.—NK activity of $Rh^+$ and $Rh^-$ melanoma patients against MM200 target cells. Mean percent $^{51}Cr$ release for $Rh^+$ and $Rh^-$ males were $13·3 \pm 6$ and $16·9 \pm 7·6$ respectively; for females, $13·8 \pm 7·0$ and $16·3 \pm 6·3$.

of NK-cell activity for each patient were then calculated and are shown as histograms in Fig. 2 for $Rh^+$ and $Rh^-$ male and female patients. The mean % $^{51}Cr$ release against the MM200 target cells for 95 $Rh^+$ males was $13·3 \pm 6$ (243 estimations) and for the 18 $Rh^-$ males $16·9 \pm 7·6$ (41 estimations) ($0·0125 < P < 0·025$). Analysis of the NK-cell values of melanoma patients at any one time showed statistically significant difference between $Rh^+$ and $Rh^-$ patients. We assume this is due to the inherent variability of the assays from day to day which was largely circumvented in the studies on normal subjects by carrying out a large number of assays on each day. Another source of extra variation in the results from melanoma patients may have been the effect of treatment with BCG and chemotherapy on NK-cell activity. Both sources of variation could be expected to obscure some of the influence of Rh antigens on NK-cell activity noted in the normal subjects.

*Absence of detectable Rh antigens on melanoma or Chang cells*

It was considered possible that the higher NK-cell activity of $Rh^-$ subjects may have been due to recognition of Rh antigens C and D on the target cells. To examine this possibility the target cells were tested for the presence of Rh antigens C and D, using the IgG fraction of antisera against these antigens in $^{51}Cr$-release LDA assays. No Rh antigens were detected on the Chang or MM200 cells by these methods nor on melanoma cell from 3 $Rh^+$ patients.

It was also considered that target cells from $Rh^+$ patients might have antigens detected by NK cells from $Rh^-$ subjects that were not detectable by serological means. To examine this possibility, the NK-cell activity of $Rh^-$ subjects was tested against melanoma target cells from both $Rh^+$ and $Rh^-$ melanoma patients. The ratios of NK-cell activity against target cells from $Rh^-$ and $Rh^+$ patients were then compared to the ratios of NK-

FIG. 3.—Cumulative survival of melanoma patients (868 male, 935 female) (a) according to sex and Rh status. $Rh^-$, ——; $Rh^+$, – – –; male, ●; female, ○; (b) In female patients, according to parity and Rh status $Rh^-$, ——; $Rh^+$, – – –; parous, ●; non parous, ○. The improved survival of $Rh^-$ (Fig. 3a) is seen to be confined to parous females.

cell activity of $Rh^+$ normal subjects against the same target cells. Ten $Rh^-$ and 10 $Rh^+$ subjects were tested against melanoma cells from 2 $Rh^+$ patients and 2 melanoma cells from $Rh^-$ patients. The ratio of NK-cell activity of $Rh^-$ subjects against target cells from $Rh^+$ and $Rh^-$ patients was not significantly different from the ratio of NK-cell activity of $Rh^+$ subjects against the same target cells. These experiments therefore did not support the idea that the higher NK-cell activity of $Rh^-$ subjects was due to sensitization against Rh antigens on the target cells.

*Comparison of the survival of $Rh^-$ and $Rh^+$ melanoma patients*

To determine whether the observed differences in NK-cell values between the $Rh^-$ and $Rh^+$ patients may also be reflected in a difference in survival of the two groups, the cumulative survival rates of all patients who attended the melanoma unit from 1963 to December 1977 (935 females and 868 males) were determined, as described by Peto *et al.* (1977). The results in Fig. 3(a) indicated a continuous trend for improved survival of $Rh^-$ females, but the reverse was found for males. Comparison of the cumulative 10-year survival rates of $Rh^-$ and $Rh^+$ women gave a $\chi^2$ value of 1·15 ($P<0{\cdot}3$). The equivalent value for the 10-year cumulative survival rates of males was $\chi^2=0{\cdot}89$ ($P<0{\cdot}5$). Further analysis of the data for females shown in Fig. 3(b) indicated that the improved survival of $Rh^-$ females applied only to parous women. The $\chi^2$ value for the comparison of $Rh^-$ and $Rh^+$ parous women was 1·95 ($P<0{\cdot}20$). These latter data are similar to our previous published data on the effect of parity on survival from melanoma (Hersey *et al.*, 1977).

### DISCUSSION

The difference in NK-cell activity shown in these studies between the $Rh^-$ and $Rh^+$ subjects of $\sim$20%, appeared to apply to both normal subjects and melanoma patients. This difference was only detectable by comparison of a large number of subjects, and indicated that Rh antigens were probably only one of several influences on the level of NK-cell activity. No association was found between NK-cell activity and the ABO blood groups nor between NK-cell activity and sex. The latter result was in contrast to that previously reported by Santoli *et al.* (1976) but we are unable to offer an explanation for this difference.

The values for melanoma patients represent the average for assays carried out at different times on patients with localized melanoma before and after surgery and on patients with disseminated melanoma. Patients in both groups received various forms of chemotherapy and immunotherapy with BCG, both of which are known to influence levels of NK-cell activity. It is therefore possible that the difference in NK-cell activity noted between $Rh^-$ and $Rh^+$ melanoma patients reflects a bias towards a particular form of treatment. Analysis of the clinical data, however, revealed no such bias in patient management. It is also known that NK-cell activity is decreased in patients with disseminated tumours (Takasugi *et al.*, 1977; Pross & Baines, 1976) but again, analysis of our data revealed no significant difference between the proportion of patients with disseminated melanoma in the $Rh^-$ and $Rh^+$ groups. These considerations of course do not apply to the studies on normal subjects.

As discussed previously, it has also been shown that the HLA-A3B7 haplotype appears to be associated with low NK-cell activity (Petranyi *et al.*, 1974; Santoli *et al.*, 1976) and it is possible that inheritance of this haplotype may have influenced our results. However, we know of no evidence that this particular HLA haplotype is preferentially associated with one or other Rh antigens.

The mechanism underlying the influence of Rh antigens on NK-cell activity is unknown. We were unable to obtain evidence of Rh antigens on the target cells used in this study, and it therefore appears unlikely that sensitization of $Rh^-$ subjects to Rh antigens on the target cells would account for the results.

In mice it was shown that NK-cell activity was linked to $H_2$ genes, and it was postulated that NK-cell activity might be partly controlled by immune-response genes in this region, analogous to those known to regulate antibody production (Kiessling & Haller, 1978). It is therefore possible that human genes regulating NK-cell activity may be associated with the genes coding for Rh antigens. This suggestion received some support from the results of a workshop on HLA and immune responses reported by Petranyi *et al.* (1974). In those studies on 133 normal subjects, it was found that $Rh^+$ subjects had high natural antibody levels and high lymphocyte responsiveness to phytohaemagglutinin, which correlated with low spontaneous lymphocyte cytotoxicity to xenogeneic target cells. In view of the recent reports of the influence of interferon on NK-cell activity (Trinchieri & Sanatoli, 1978) it may also be possible that genes coding for Rh antigens may be associated with genes regulating interferon production.

In our analysis of the survival rates of $Rh^-$ and $Rh^+$ patients, we hoped to determine whether the difference in NK-cell activity in these two populations affected their survival rates. This analysis was prompted by a number of studies in experimental animals which suggested that NK-cell activity may be important in the host's defence against tumours *in vivo* (Kiessling & Haller, 1978).

Our results were conflicting, in that while $Rh^-$ females had apparently better survival rates than $Rh^+$ females, the reverse was found for males. These data therefore do not support a role for NK-cell activity in protection against established melanoma via the higher NK-cell activity for $Rh^-$ subjects in both males and females. There are, however, a number of limitations in using these data to determine whether NK-cell activity has a role *in vivo* against melanoma; thus the 20% difference in NK-cell activity between the two populations may be too small to be reflected in gross survival rates. Alternatively, Rh antigens may be associated with the expression of other immune-response genes, such as those coding for antibody production, which may have an opposing and greater influence on survival than that of NK-cell activity.

Similar considerations also apply to the role of NK-cell activity in preventing the onset of melanoma, in that if NK-cell activity has a role in surveillance $Rh^-$ subjects would be expected to have a lower incidence of melanoma than $Rh^+$ subjects. The ratio of $Rh^-$ to $Rh^+$ patients in our series was, however, not significantly different from that in the normal population. Again, these data do not support a role for NK-cell activity in preventing the onset of melanoma, but the objections to using the data in this way are as discussed above.

Although our results suggest that Rh antigens are associated with NK-cell activity, this is not reflected in the survival rates of our melanoma patients. The main importance of our results is the suggestion that Rh antigens may be linked to genes which regulate the activity of these cytotoxic cells. It is unlikely that the Rh antigens are directly involved in this regulation, since they have so far only been detected on red blood cells, and it seems more likely therefore that the $Rh^-$ haplotype is associated in some way with genes regulating NK-cell activity.

This work was supported by the NSW State Cancer Council and in part by the National Cancer Institute Contract No. 1-CB-74120.

We wish to thank nursing sisters J. Seggie, R. Frew and R. Brissenden for their help in collecting clinical specimens.

### REFERENCES

Akira, D. & Takasugi, M. (1977) Loss of specific natural cell-mediated cytotoxicity with absorption of natural antibodies from serum. *Int. J. Cancer*, **19**, 747.

Bonnard, G. D., Kay, D. H., Herberman, R. B., & 4 others (1979) Models for the mechanism of natural cell-mediated cytotoxicity. 1. Relationship to antibody dependent cell-mediated cytotoxicity. In *Prospectives in Immunology*, Ed. H. Reithmuller, P. Wernet & H. Cudkowicz. New York: Acad. Press. (In Press).

BÖYUM, H. (1968) Isolation of mononuclear cells and granulocytes from human blood. *Scan. J. Clin. Lab. Invest.*, **21**, 79.

HERSEY, P. (1979) Natural killer cells—A new cytotoxic mechanism against tumours. *Aust. N.Z. J. Med.* (In Press).

HERSEY, P., EDWARDS, A. E., EDWARDS, J., ADAMS, E., MILTON, G. W. & NELSON, D. S. (1975) Specificity of cell-mediated cytotoxicity against human melanoma lines. Evidence for non specific killing by activated T cells. *Int. J. Cancer*, **16**, 173.

HERSEY, P., EDWARDS, A. E. & EDWARDS, J. (1976) Characterization of effector cells in human blood. *Clin. Exp. Immunol*, **23**, 104.

HERSEY, P., MORGAN, G., STONE, D., MCCARTHY, W. H. & MILTON, G. W. (1977) Prior pregnancy as a protective factor against death from melanoma. *Lancet*, i, 451.

HERSEY, P., EDWARDS, A., MILTON, G. W. & MCCARTHY, W. H. (1978) Relationship of cell-mediated cytotoxicity against melanoma cells to prognosis in melanoma patients. *Br. J. Cancer*, **37**, 505.

KIESSLING, R., PETRANYI, G., KLEIN, G. & WIGZELL, H. (1975) Genetic variation of *in vitro* cytotoxic activity and *in vivo* rejection potential of non-immunized semi-syngeneic mice against a mouse lymphoma line. *Int. J. Cancer*, **15**, 933.

KIESSLING, R. & HALLER, O. (1978) Natural killer cells in the mouse: an alternative immune surveillance mechanism? In *Contemporary Topics in Immunology*, Ed. N. L. Warner, **8**, 171.

PETER, H. H., EIFE, R. F. & KALDEN, J. R. (1976) Spontaneous cytotoxicity (SCMC) of normal human lymphocytes against a human melanoma cell line. A phenomenon due to a lymphotoxin-like mediator. *J. Immunol.*, **116**, 342.

PETO, R., PIKE, M. C., ARMITAGE, P. & 7 others (1977) Design and analysis of randomized clinical trials requiring prolonged observation of each patient. *Br. J. Cancer*, **35**, 1.

PETRANYI, G. G., IVANYI, P. & HOLLAN, S. R. (1974) Relations of HL-A and Rh systems to immune reactivity joint report of the results of HL-A and immune response workshop, Budapest, 1972. *Vox. Sang.*, **27**, 470.

PROSS, H. F. & BAINES, M. G. (1976) Spontaneous human lymphocyte-mediated cytotoxicity against tumour target cells. 1. The effect of malignant disease. *Int. J. Cancer*, **18**, 593.

PROSS, H. F. & BAINES, M. G. (1978) Spontaneous human lymphocyte mediated cytotoxicity against tumour target cells. *Cancer Immunol. Immunother.*, **3**, 75.

SANTOLI, D., TRINCHIERI, G., ZMIJEWSKI, C. M. & KOPROWSKI, H. (1976) HLA-related control of spontaneous and antibody-dependent cell-mediated cytotoxic activity in humans. *J. Immunol.*, **117**, 765.

TAKASUGI, M., RAMSEYER, A. & TAKASUGI, J. (1977) Decline of natural non-selective cell mediated cytotoxicity in patients with tumour progression. *Cancer Res.*, **37**, 413.

TRINCHIERI, G. & SANTOLI, D. (1978) Antiviral activity induced by culturing lymphocytes with tumour-derived or virus transformed cells. Enhancement of human natural killer cell activity by interferon and antagonistic inhibition of susceptibility of target cells to Lysis. *J. Exp. Med.*, **147**, 1314.

WEST, H. W., CANNON, G. B., KAY, D., BONNARD, G. D. & HERBERMAN, R. B. (1977) Natural cytotoxic reactivity of human lymphocytes against a myeloid cell line: characterization of effector cells. *J. Immunol.*, **118**, 355.


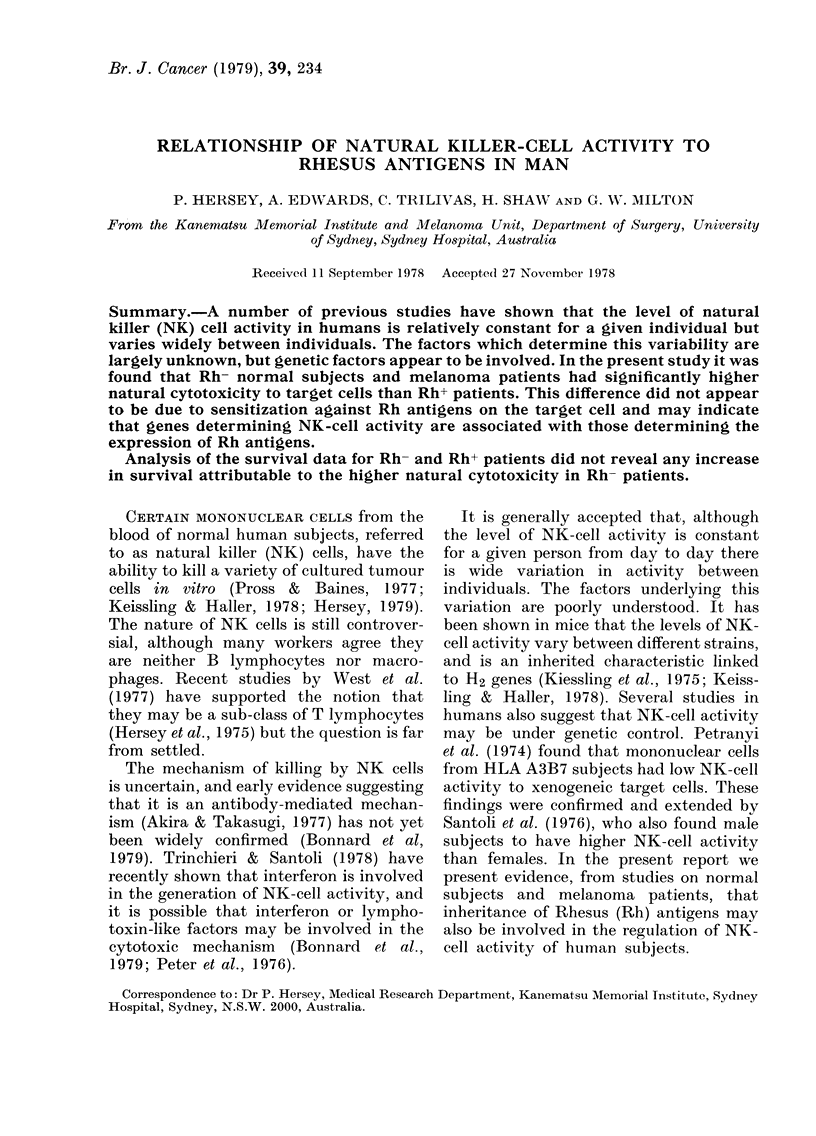

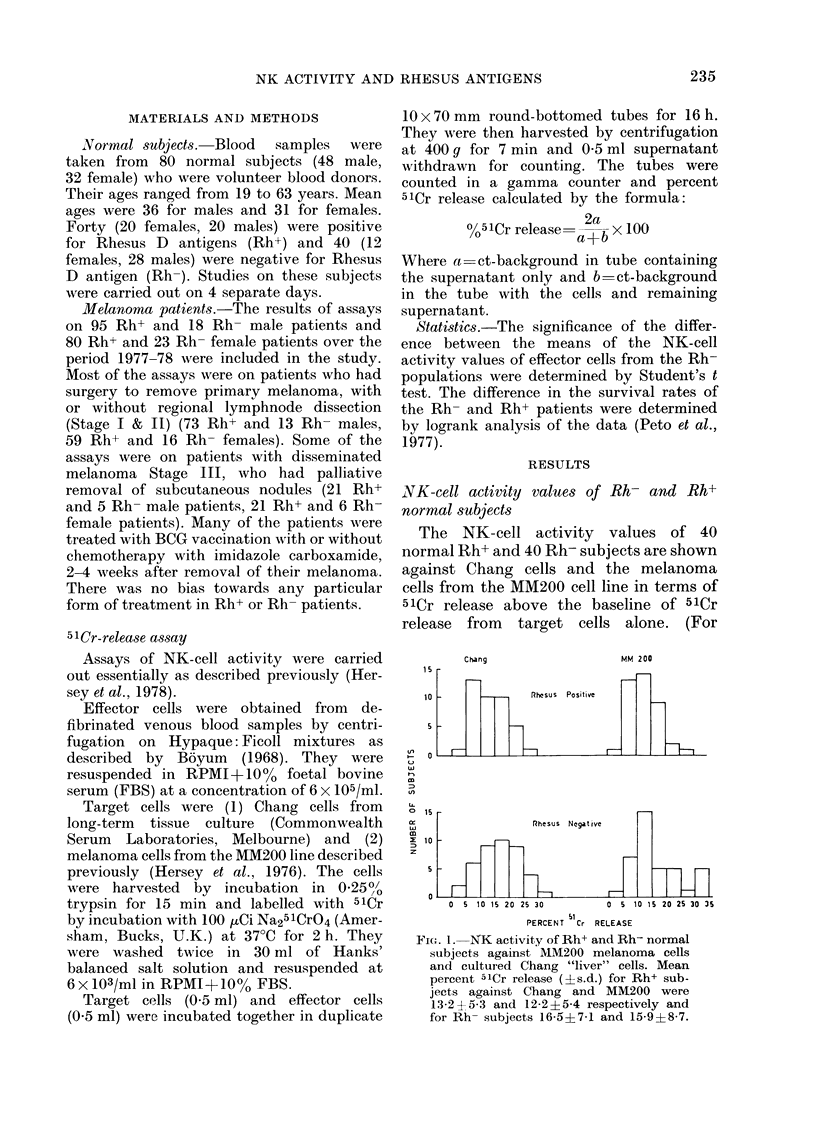

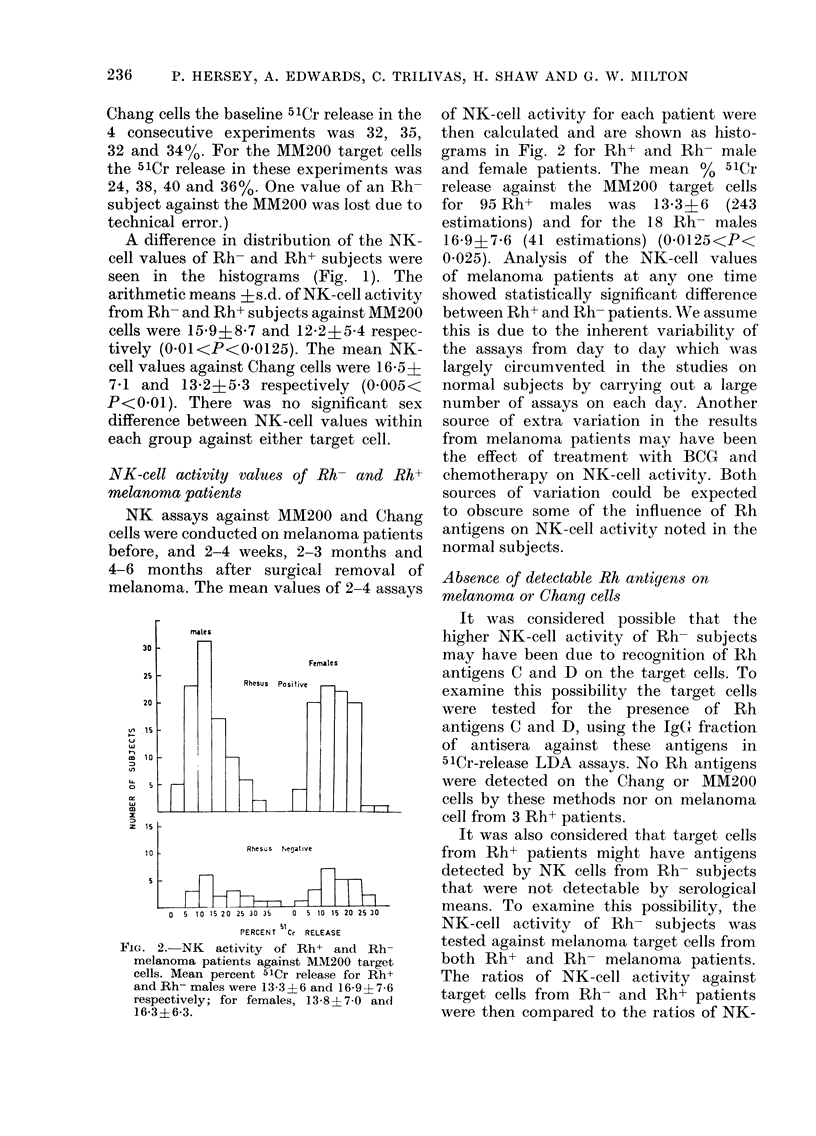

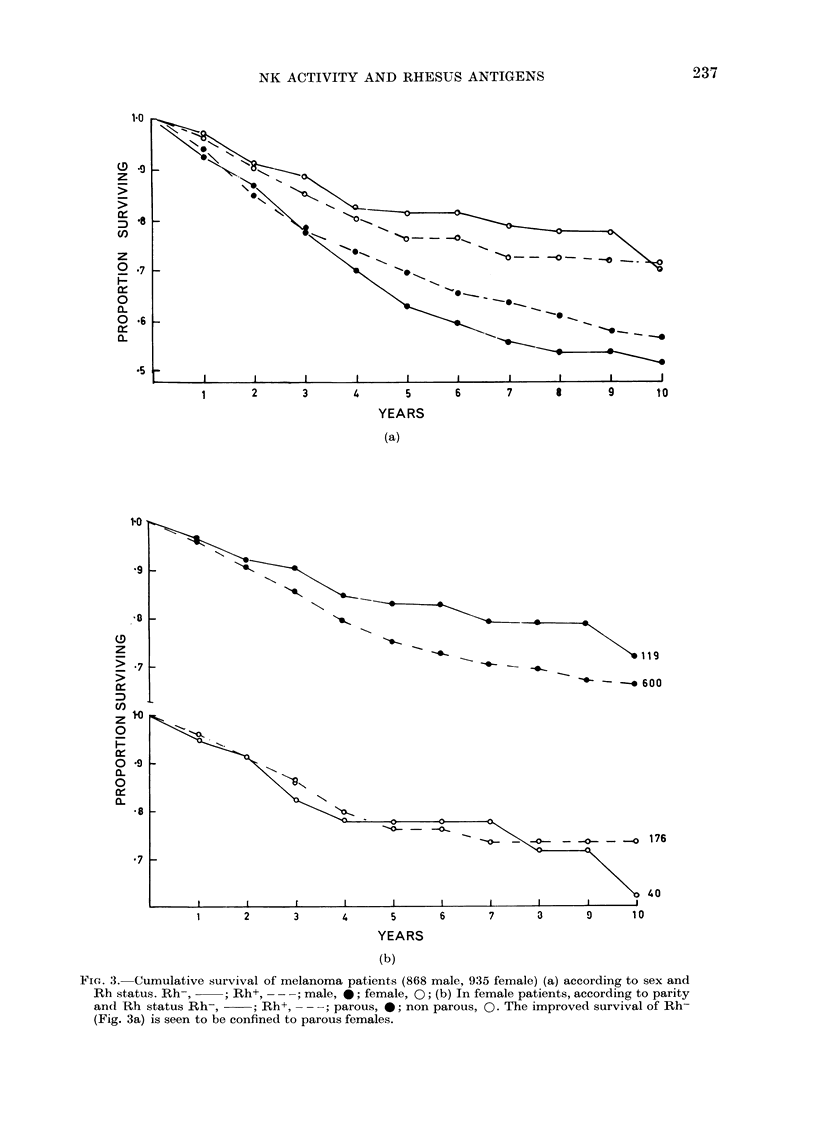

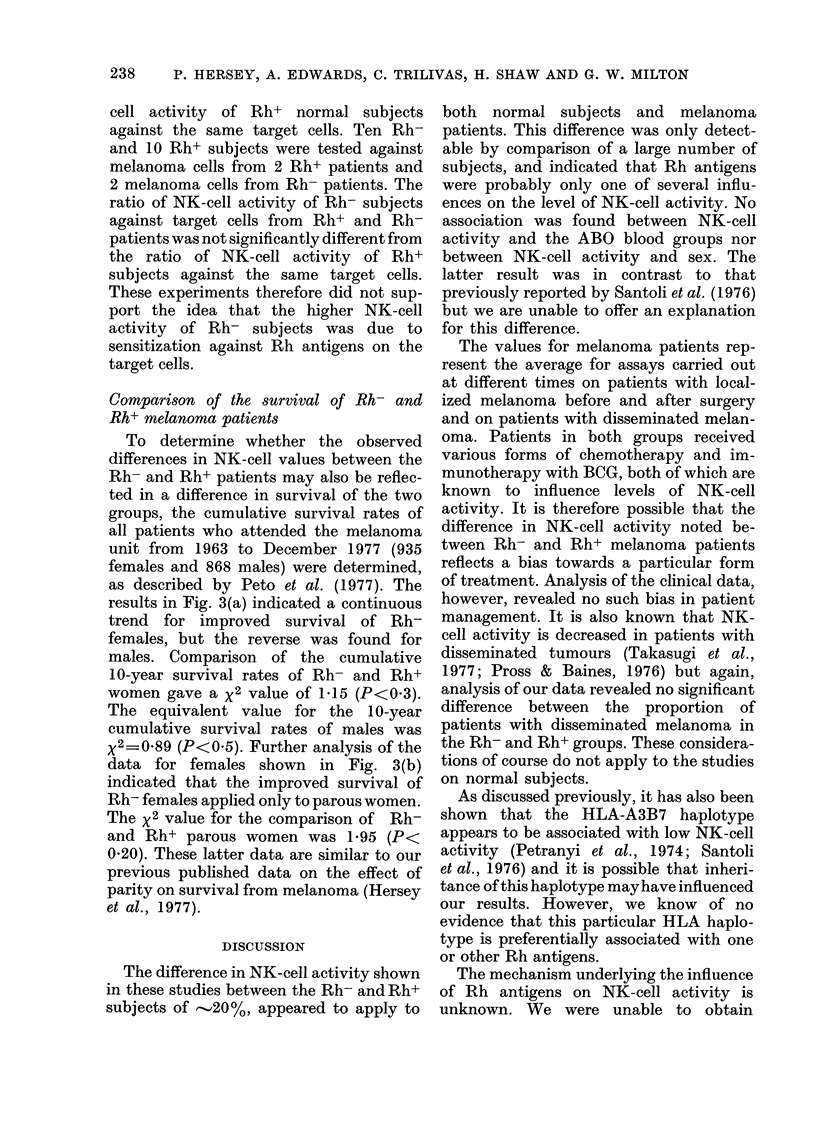

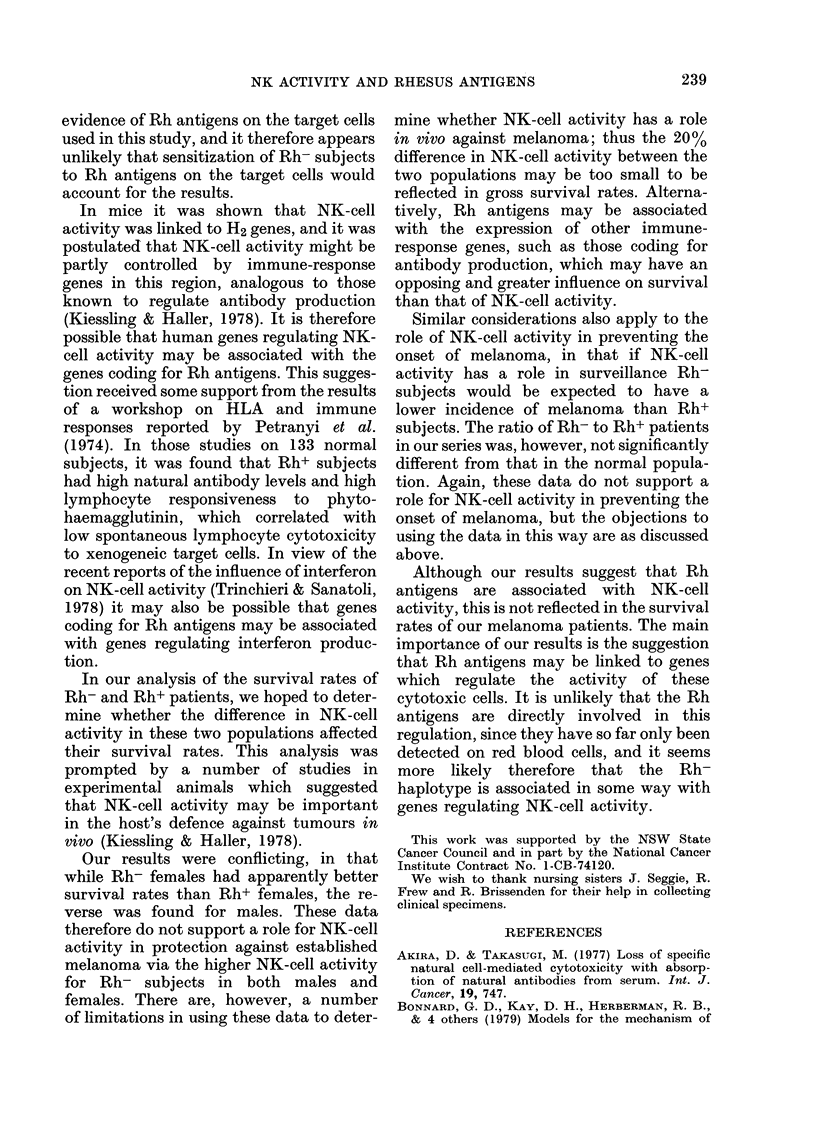

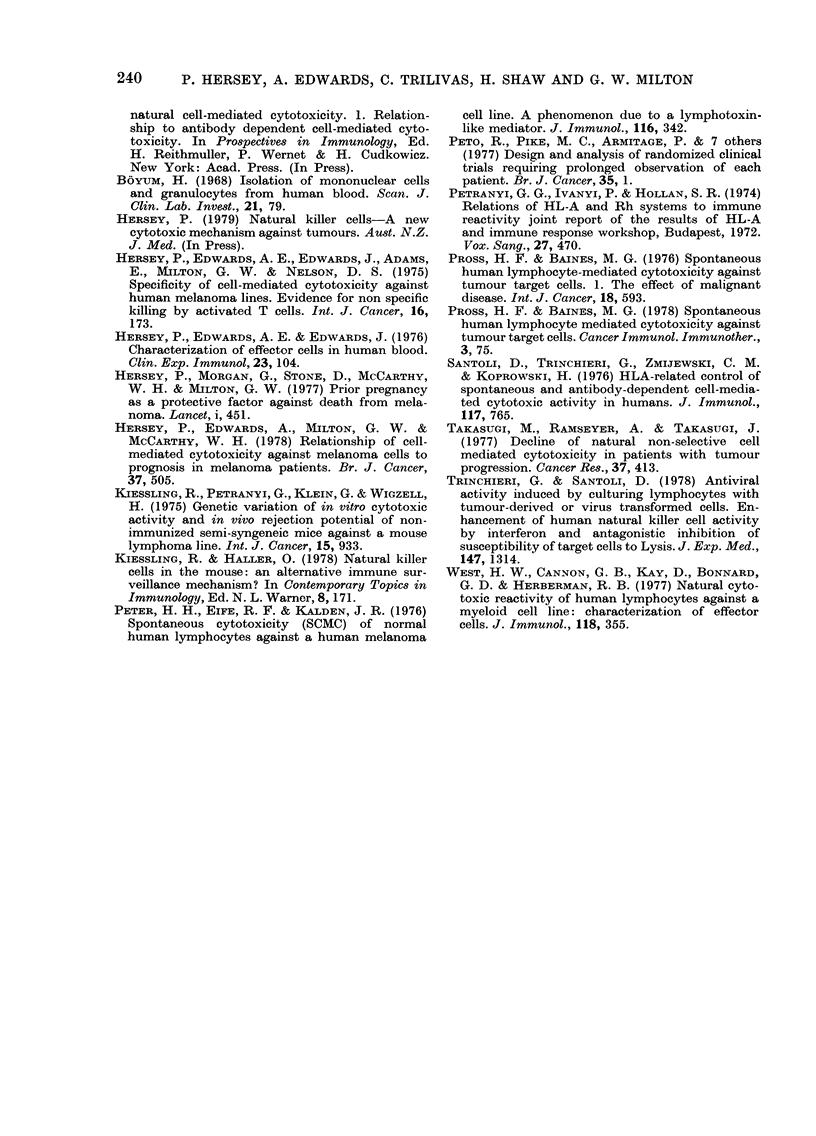


## References

[OCR_00672] Akira D., Takasugi M. (1977). Loss of specific natural cell-mediated cytotoxicity with absorption of natural antibodies from serum.. Int J Cancer.

[OCR_00700] Hersey P., Edwards A., Edwards J., Adams E., Milton G. W., Nelson D. S. (1975). Specificity of cell-mediated cytotoxicity against human melanoma lines: evidence for "non-specific" killing by activated T-cells.. Int J Cancer.

[OCR_00708] Hersey P., Edwards A., Edwards J. (1976). Characterization of mononuclear effector cells in human blood.. Clin Exp Immunol.

[OCR_00713] Hersey P., Morgan G., Stone D. E., McCarthy W. H., Milton G. W. (1977). Previous pregnancy as a protective factor against death from melanoma.. Lancet.

[OCR_00719] Hershey P., Edwards A., Milton G. W., McCarthy W. H. (1978). Relationship of cell-mediated cytotoxicity against melanoma cells to prognosis in melanoma patients.. Br J Cancer.

[OCR_00733] Kiessling R., Haller O. (1978). Natural killer cells in the mouse: an alternative immune surveillance mechanism?. Contemp Top Immunobiol.

[OCR_00726] Kiessling R., Petranyi G., Klein G., Wigzel H. (1975). Genetic variation of in vitro cytolytic activity and in vivo rejection potential of non-immunized semi-syngeneic mice against a mouse lymphoma line.. Int J Cancer.

[OCR_00739] Peter H. H., Eife R. F., Kalden J. R. (1976). Spontaneous cytotoxicity (SCMC) of normal human lymphocytes against a human melanoma cell line: a phenomenon due to a lymphotoxin-like mediator.. J Immunol.

[OCR_00760] Pross H. F., Baines M. G. (1976). Spontaneous human lymphocyte-mediated cytotoxicity againts tumour target cells. I. The effect of malignant disease.. Int J Cancer.

[OCR_00772] Santoli D., Trinchieri G., Zmijewski C. M., Koprowski H. (1976). HLA-related control of spontaneous and antibody-dependent cell-mediated cytotoxic activity in humans.. J Immunol.

[OCR_00779] Takasugi M., Ramseyer A., Takasugi J. (1977). Decline of natural nonselective cell-mediated cytotoxicity in patients with tumor progression.. Cancer Res.

[OCR_00785] Trinchieri G., Santoli D. (1978). Anti-viral activity induced by culturing lymphocytes with tumor-derived or virus-transformed cells. Enhancement of human natural killer cell activity by interferon and antagonistic inhibition of susceptibility of target cells to lysis.. J Exp Med.

[OCR_00794] West W. H., Cannon G. B., Kay H. D., Bonnard G. D., Herberman R. B. (1977). Natural cytotoxic reactivity of human lymphocytes against a myeloid cell line: characterization of effector cells.. J Immunol.

